# Supplementary material for: Assessment of environmental impacts and costs for hydrogen-powered cargo shipping in Europe
Source: Commun Eng. 2025 Mar 17;4:50. doi: 10.1038/s44172-025-00388-4 (PMC11914104; doi:10.1038/s44172-025-00388-4)
Supplement: Supplementary file 2 — Supplementary Materials [file 44172_2025_388_MOESM2_ESM.pdf]

Supplementary Materials: Assessment of environmental impacts and costs for hydrogen-powered cargo shipping in Europe

Simon Schlehuber[1,*], Moritz Gutsch[1], Niklas Kronemeyer[1,2], Florian Frieden[1,3], Stephan von Delft[4,5]

[1] Institute of Business Administration at the Department of Chemistry and Pharmacy, University of Münster, Leonardo Campus 1, 48148 Münster, Germany

[2] Porsche Consulting GmbH, Siemensstraße 6, 70469 Stuttgart, Germany

[3] FutureCamp Climate GmbH, Aschauer Straße 30, 81549 München, Germany

[4] University of Münster, REACH EUREGIO Start-up Center, Geiststrasse 24, 48151 Münster, Germany

[5] University of Glasgow, Adam Smith Business School, Glasgow, G12 8QQ, United Kingdom

*Corresponding author. E-mail address: simon.schlehuber@uni-muenster.de

Supplementary Note 1: Boat design and calculation of relevant drag force parameters

As described in the main section, the autonomous-driving hydrogen-powered boat (AHB) is 20 m long, 6 m wide and 1.5 m deep. It therefore has enough space to carry one forty-foot equivalent unit (FEU) container while simultaneously allowing for all required auxiliaries like the fuel cell stacks, the hydrogen storage and the electric motors. The two hulls are modelled as triagonal prisms so that the total weight of aluminum needed for constructing the AHB can more easily be estimated. Both the deck and the hulls are 1.5 cm thick, making the hulls hollow on the inside. The total volume of the hulls is easily large enough to support one fully loaded FEU container (maximum allowed weight of 30,480 kg[1]).

To make sure that the total cost of ownership (TCO) and life cycle assessment (LCA) results of the AHB are reasonable and therefore usable for comparison to data from literature, it is important to have a boat design that is not completely out of touch with reality. In the proposed TCO- and LCA-analysis, Eq. (S1) (also see Eq. 5 of manuscript) plays a key role because it determines the power needed to propel the model boat forward at different speeds. The necessary power of the drivetrain, in turn, greatly

impacts final costs and global warming potential since elements of adequate power need to be installed and fuel requirements will change accordingly. Next to speed, Eq. (S1) includes the two boat-specific parameters wetted surface area and total drag coefficient (A and c_T) which also have a major influence on the TCO- and LCA-outcome. This was confirmed by the sensitivity analysis (see Figure 4 of manuscript).

$$\mathrm{Power}_{\mathrm{Fuel\ Cell\ Stack;\ Sustain\ Cruise\ Speed}} = F_{\mathrm{Drag,\ boat}} \cdot v_{\mathrm{R}} = 0.5 \cdot \rho \cdot \mathrm{v_R}^3 \cdot c_{\mathrm{T}} \cdot A \qquad \text{(S1)}$$

Therefore, we decided to address these two parameters with more caution by carrying out a rigorous derivation based on work by Molland[2]. Table S1 provides an overview of the most important parameters and their calculation for the determination of A and c_T.

Table S1: Relevant parameters for the calculation of wetted surface area A and total drag coefficient c_T.

Description	Value	Equation
Length of AHB: L	20 m	*Assumption*
Breadth of AHB: B	6 m	*Assumption*
Breadth of one hull: b	1.9 m	*Assumption*
Depth of AHB: T	1.5	*Assumption*
Displaced volume per hull: V_D	25 m^3	*Assumption*
Max speed of AHB (scenario 2): v_{max}	4.17 m/s	*Assumption*
$L/V_D^{1/3}$	6.84	$L/V_{\mathrm{D}}^{1/3}$
Separation between catamaran demihull centerlines: S_c	4.1 m	$B - 2 \cdot \frac{b}{2}$
S_C/L	0.205	S_c/L
L/b	10.53	L/b
b/T	1.27	b/T
Block coefficient per hull (c_B)	0.439	$\frac{V_D}{L \cdot b \cdot T}$
Wetted surface coefficient (c_s)	2.645	Linear regression: see Table 10.20 and Eq. 10.89 in [2] (2011)
Wetted surface area of one hull (A_{Hull})	59.15 m^2	$c_S \cdot \sqrt{V_D \cdot L}$
**Wetted surface area of AHB (A)**	**118.31 m^2**	$\mathbf{2 \cdot A_{Hull}}$
Froude number (Fr) with g = 9.81 m/s^2	0.297	$\frac{v}{\sqrt{g \cdot L}}$
Coefficient of residuary resistance (c_R)	0.0035	Based on multiple linear regressions and equations in Molland[2], see Section 10.3.5.1

		and Table A3.26 of the appendix
Coefficient of frictional resistance for the AHB (c_F)	0.0022	Based on comparison to Southampton series model boat, see Section 17.2.10 and Eq. 10.61 in Molland[2]
Coefficient of frictional resistance for the Southampton series model boat needed for final calculation (c_{FM})	0.0042	see Section 17.2.10 and Eq. 10.61 in Molland[2]
Form factor for catamarans (1+ßk)	1.404	$3.03 \cdot \left(\frac{L}{V_D^{1/3}}\right)^{-0.4}$
Residuary resistance factor τ_R	1.286	Based on multiple linear regressions and equations in Molland[2], see Section 10.3.5.1 and Table A3.26 of the appendix
**Total drag coefficient (c_T)**	**0.0059**	$\boldsymbol{c_F + \tau_R c_R - ßk \cdot (c_{FM} - c_F)}$

For more background information regarding the calculation of all parameters, the reader is referred to sections 10.3.5, 10.4.3, 17.2.10, and Appendix A3 of Molland[2] (2011).

Supplementary Note 2: LCA results for all 16 impact categories

Because a full LCA features more than just the impact category of climate change, this section provides the results for all 16 impact categories. The results were determined by using the life cycle inventory assessment (LCIA) characterization model Environmental Footprint 3.1[3]. First, Table S2 provides an overview of the 16 impact categories.

Table S2: Overview of the 16 impact categories of the Environmental Footprint 3.1 LCIA characterization model.

Impact category	Indicator	Unit
acidification	accumulated exceedance (AE)	mol H+ eq.
climate change	global warming potential (GWP100)	kg CO_2 eq.
ecotoxicity: freshwater	comparative toxic unit for ecosystems (CTUe)	CTUe
energy resources: non-renewable	abiotic depletion potential (ADP): fossil fuels	MJ, net calorific value

eutrophication: freshwater	fraction of nutrients reaching freshwater end compartment (P)	kg P eq.
eutrophication: marine	fraction of nutrients reaching marine end compartment (N)	kg N eq.
eutrophication: terrestrial	fraction of nutrients reaching marine end compartment (N)	mol N eq.
human toxicity: carcinogenic	comparative toxic unit for human (CTUh)	CTUh
human toxicity: non-carcinogenic	comparative toxic unit for human (CTUh)	CTUh
ionising radiation: human health	human exposure efficiency relative to U235	kBq U235 eq.
land use	soil quality index	dimensionless
material resources: metals/minerals	abiotic depletion potential (ADP): elements (ultimate reserves)	kg Sb eq.
ozone depletion	ozone depletion potential (ODP)	kg CFC-11 eq.
particulate matter formation	impact on human health	disease incidence
photochemical oxidant formation: human health	tropospheric ozone concentration increase	kg NMVOC eq.
water use	user deprivation potential (deprivation-weighted water consumption)	m^3 world eq. deprived

The results for each impact category when the model AHB is powered by green hydrogen are shown in Table S3.

Table S3: Results of the LCIA for each impact category when AHB is powered by green hydrogen.

Impact category [unit]	Scenario 1 (5 km/h)	Scenario 2 (10 km/h)	Scenario 3 (15 km/h)	Scenario 4 (20 km/h)
acidification [mol H+ eq. per km]	0.0025	0.0027	0.0040	0.0060
climate change [kg CO2eq. per km]	0.3464	0.3284	0.4668	0.6968
ecotoxicity: freshwater [CTUe per km]	1.7681	2.4207	4.0864	6.5333
energy resources: non-renewable [MJ per km]	3.8012	3.8760	5.6761	8.5440
eutrophication: freshwater [kg P eq. per km]	0.0001	0.0002	0.0003	0.0004
eutrophication: marine [kg N eq. per km]	0.0004	0.0004	0.0006	0.0009

eutrophication: terrestrial [mol N eq. per km]	0.0042	0.0043	0.0063	0.0095
human toxicity: carcinogenic [CTUh per km]	4.8804E-10	5.4575E-10	8.6063E-10	1.3497E-09
human toxicity: non-carcinogenic [CTUh per km]	9.5074E-09	1.3149E-08	2.2608E-08	3.6588E-08
ionizing radiation: human health [kBq U235 eq. per km]	0.0195	0.0236	0.0360	0.0543
land use [per km]	8.5033	20.0812	39.7331	67.3197
material resources: metals/minerals [kg Sb eq. per km]	5.3738E-06	1.1357E-05	2.1875E-05	3.6696E-05
ozone depletion [kg CFC-11 eq. per km]	1.8605E-08	3.1153E-08	5.2053E-08	8.0226E-08
particulate matter formation [disease incidence per km]	2.6857E-08	2.5492E-08	3.6614E-08	5.5112E-08
photochemical oxidant formation: human health [kg NMVOC eq. per km]	0.0013	0.0014	0.0021	0.0032
water use [m^3 world eq. deprived per km]	0.1596	0.2536	0.4546	0.7453

The results for each impact category when the model AHB is powered by gray hydrogen are shown in Table S4.

Table S4: Results of the LCIA for each impact category when AHB is powered by gray hydrogen.

Impact category [unit]	Scenario 1 (5 km/h)	Scenario 2 (10 km/h)	Scenario 3 (15 km/h)	Scenario 4 (20 km/h)
acidification [mol H+ eq. per km]	0.0021	0.0017	0.0021	0.0028
climate change [kg CO2eq. per km]	0.5015	0.7160	1.2419	2.0144
ecotoxicity: freshwater [CTUe per km]	1.2651	1.1632	1.5713	2.2576
energy resources: non-renewable [MJ per km]	6.5894	10.8465	19.6170	32.2435
eutrophication: freshwater [kg P eq. per km]	1.0367E-04	7.2954E-05	7.7572E-05	9.5596E-05
eutrophication: marine [kg N eq. per km]	0.0004	0.0003	0.0004	0.0005
eutrophication: terrestrial [mol N eq. per km]	0.0037	0.0032	0.0041	0.0058

human toxicity: carcinogenic [CTUh per km]	3.7376E-10	2.6005E-10	2.8923E-10	3.7831E-10
human toxicity: non-carcinogenic [CTUh per km]	5.8825E-09	4.0869E-09	4.4830E-09	5.7759E-09
ionizing radiation: human health [kBq U235 eq. per km]	0.0154	0.0135	0.0158	0.0199
land use [per km]	0.6918	0.5525	0.6756	0.9219
material resources: metals/minerals [kg Sb eq. per km]	1.5123E-06	1.7031E-06	2.5669E-06	3.8728E-06
ozone depletion [kg CFC-11 eq. per km]	2.5484E-08	4.8349E-08	8.6445E-08	1.3869E-07
particulate matter formation [disease incidence per km]	2.2252E-08	1.3981E-08	1.3592E-08	1.5973E-08
photochemical oxidant formation: human health [kg NMVOC eq. per km]	0.0014	0.0015	0.0024	0.0037
water use [m^3 world eq. deprived per km]	0.0822	0.0601	0.0676	0.0874

Supplementary Note 3: Marginal abatement cost analysis

Marginal abatement cost (MAC) analysis is a method used to quantify the economic trade-offs involved in reducing emissions. It provides insight into how much it costs to decrease the carbon footprint of a product by 1 kg CO_2 eq. As such, it also enables a comparison between the AHB and the reference diesel truck[4]. Because the reference truck is solely analyzed on a TCO basis, GWP100 values for the diesel truck are taken from the average CO_2 emissions of diesel trucks (type 5-LH) in the EU[5]. The results for all MACs of the AHB are determined against the diesel truck. Table S5 shows the raw data for scenario 2 (10 km/h) as well as final MACs. Table S6 shows the raw data for scenario 3 (15 km/h) as well as final MACs.

Table S5: Raw data and MACs for the scenario 2 (10 km/h) AHB powered by either green or gray hydrogen against the reference truck.

Type of transport	TCO [€/km]	GWP100 [kg CO_2 eq/km]
Diesel Truck	0.652	0.784
AHB: green H_2	0.576	0.328
AHB: gray H_2	0.338	0.716

MAC vs. diesel truck	MAC [€/kg CO_2 eq]
AHB: green H_2	-0.167
AHB: gray H_2	-4.641

Table S6: Raw data and MACs for the scenario 3 (15 km/h) AHB powered by either green or gray hydrogen against the reference truck.

Type of transport	TCO [€/km]	GWP100 [kg CO_2 eq/km]
Diesel Truck	0.652	0.784
AHB: green H_2	0.957	0.467
AHB: gray H_2	0.556	1.242
**MAC vs. diesel truck**	**MAC [€/kg CO_2 eq]**	
AHB: green H_2	0.964	
AHB: gray H_2	0.210	

Supplementary Note 4: Parameters for the TCO analysis

To provide more transparency, Table S7 shows the values of parameters utilized during the calculation of the TCO for the different scenarios.

Table S7: Overview of utilized parameters for the TCO-model. Sources for the values are provided unless we calculated or assumed the value of the parameter.

Parameter	v_{GPS} [km/h]	v_{River} [km/h]	$v_{R,\ min}$ [km/h]	$v_{R,\ max}$ [km/h]	c_T	A [m^2]
Scenario 1	5	5	0	10	0.0059	118.31
Scenario 2	10	5	5	15	0.0059	118.31
Scenario 3	15	5	10	20	0.0059	118.31
Scenario 4	20	5	15	25	0.0059	118.31
**Parameter**	$Efficiency_{Fuel\ Cell}$[6]	$Efficiency_{E\text{-}Motor}$[7]	$c_{Hydrogen\ fuel\ cell,\ specific}$ [€/kW][8]	$c_{E\text{-}Motor,\ specific}$ [€/kW][9]	Total hours of operation [h]	$c_{Green\ Hydrogen}$ [€/kg][4]
Scenario 1	0.65	0.9	150	10	5110	7
Scenario 2	0.65	0.9	150	10	5110	7
Scenario 3	0.65	0.9	150	10	5110	7
Scenario 4	0.65	0.9	150	10	5110	7
**Parameter**	$c_{Gray\ Hydrogen}$ [€/kg][10]	$c_{Loading}$ [€/trip]	Distance of one trip [km][4]	Loading frequency per year	Interest rate i	$Lifespan_{Hull}$ [years][11]
Scenario 1	2	100	805	31.7	0.05	30
Scenario 2	2	100	805	63.5	0.05	30
Scenario 3	2	100	805	95.2	0.05	30
Scenario 4	2	100	805	127.0	0.05	30

**Parameter**	$Lifespan_{Fuel\ Cell}$ [years][12,13]	$Lifespan_{E\text{-}Motor}$ [years][14,15]	$Lifespan_{Storage}$ [years][16,17]	c_{Hull} [€]	$c_{Storage,\ specific}$ [€/kWh][18]	Maintenance
Scenario 1	5	15	30	30000	15	0.05
Scenario 2	5	15	30	30000	15	0.05
Scenario 3	5	15	30	30000	15	0.05
Scenario 4	5	15	30	30000	15	0.05
**Parameter**	$C_{Al\ recycling,\ specific}$ [€/ton][19]	$Lifespan_{Boat}$ [years]	Scrap weight [kg]			
Scenario 1	1175	30	11575			
Scenario 2	1175	30	13134			
Scenario 3	1175	30	15684			
Scenario 4	1175	30	19436			

Supplementary Note 5: Data for the comparison of model AHB to different trucks

The following tables provide supplementary information for the comparison of our model AHB to different trucks. In a first step, the standard goods classification for transport statistics is clustered into two categories, one being time-sensitive classifications and the other being non-time sensitive classifications (Table S8). The clustering is carried out manually.

Table S8: Results for the clustering of the standard goods classification for transport statistics. The classifications were clustered into a time-sensitive and a non-time sensitive category. The standard goods classification for transport statistics were derived from eurostat[20].

**Time-sensitive classifications**	**Non-time sensitive classifications**
GT01: Products of agriculture, hunting, and forestry; fish and other fishing products	GT02: Coal and lignite; crude petroleum and natural gas
GT04: Food products, beverages, and tobacco	GT03: Metal ores and other mining and quarrying products; peat; uranium and thorium
GT15: Mail, parcels	GT05: Textiles and textile products; leather and leather products
GT17: Goods moved in the course of household and office removals; baggage and articles accompanying travelers; motor vehicles being moved for repair; other non-market goods n.e.c.	GT06: Wood and products of wood and cork (except furniture); articles of straw and plaiting materials; pulp, paper and paper products; printed matter and recorded media

GT18: Grouped goods: a mixture of types of goods which are transported together	GT07: Coke and refined petroleum products
GT19: Unidentifiable goods: goods which for any reason cannot be identified and therefore cannot be assigned to groups 01-16.	GT08: Chemicals, chemical products, and man-made fibers; rubber and plastic products; nuclear fuel
GT20: Other goods n.e.c.	GT09: Other non-metallic mineral products
Unknown	GT10: Basic metals; fabricated metal products, except machinery and equipment
	GT11: Machinery and equipment n.e.c.; office machinery and computers; electrical machinery and apparatus n.e.c.; radio, television and communication equipment and apparatus; medical, precision and optical instruments; watches and clocks
	GT12: Transport equipment
	GT13: Furniture; other manufactured goods n.e.c.
	GT14: Secondary raw materials; municipal wastes and other wastes
	GT16: Equipment and material utilized in the transport of goods

Utilizing these clusters and the corresponding trucking market sizes provided in billion ton kilometers[21], the following market breakdown is received (Table S9).

Table S9: Breakdown of the 2022 trucking market of the EU27 countries into the six segments. This includes the time sensitive and non-time sensitive categories as well as three different sets of trip distances.

**Distance per trip**	**Time-sensitive market segment [share of overall EU27 trucking market, in %]**	**Non-time sensitive market segment [share of overall EU27 trucking market, in %]**
1 to 499 km	26.02%	31.40%
500 to 999 km	12.03%	11.52%
1000 + km	9.47%	9.55%

In a last step, the cost assessment of the model AHB (for scenario 2, green hydrogen) in relation to potential future semi-trucks powered by diesel, battery (BET), or hydrogen fuel cell (FCET) is carried out by comparing the TCO at different average trip distances (Table S10). The data for the semi-trucks is based on work by the National Renewable Energy Laboratory (NREL)[4].

Table S10: Cost assessment of model AHB in relation to future semi-trucks powered by diesel, battery, or hydrogen fuel cell for select trip distances. For the model AHB, the TCO is calculated for scenario 2 and green hydrogen. Data for the semi-trucks is taken from the 2025 weight-constrained analysis of the NREL[4]. TCO values for trip distances of 483 km (300 miles), 805 km (500 miles), and 1207 km (750 miles) were directly provided by the NREL paper and thus utilized for linear regression.

**Scenarios (trip distance, type of truck)**	**TCO of NREL trucks [€/km]**	**TCO of model AHB [€/km]**
100 km, Diesel (NREL)	0.5158	1.4073
100 km, FCET (NREL)	0.587	1.4073
100 km, BET (NREL)	0.3912	1.4073
300 km, Diesel (NREL)	0.5558	0.7532
300 km, FCET (NREL)	0.647	0.7532
300 km, BET (NREL)	0.4712	0.7532
483 km, Diesel (NREL)	0.58	0.6384
483 km, FCET (NREL)	0.69	0.6384
483 km, BET (NREL)	0.55	0.6384
500 km, Diesel (NREL)	0.5958	0.6324
500 km, FCET (NREL)	0.707	0.6324
500 km, BET (NREL)	0.5512	0.6324
750 km, Diesel (NREL)	0.6458	0.5815
750 km, FCET (NREL)	0.782	0.5815
750 km, BET (NREL)	0.6512	0.5815
805 km, Diesel (NREL)	0.6519	0.5758
805 km, FCET (NREL)	0.82	0.5758
805 km, BET (NREL)	0.70	0.5758
1000 km, Diesel (NREL)	0.6958	0.5638
1000 km, FCET (NREL)	0.857	0.5638
1000 km, BET (NREL)	0.7512	0.5638
1207 km, Diesel (NREL)	0.7102	0.5597
1207 km, FCET (NREL)	0.91	0.5597
1207 km, BET (NREL)	0.86	0.5597

Moreover, a manual break-even analysis is conducted to find the break-even points of the model AHB's TCO compared to the TCO of diesel trucks (Table S11) as well as trucks powered by zero-emission technologies (better value of BET and FCET, Table S12).

Table S11: Break-even analysis for the TCO of the model AHB and trucks powered by diesel. For the model AHB, the TCO is calculated for scenario 2 and green hydrogen. Data for the semi-trucks is taken from the 2025 weight-constrained analysis of the NREL[4].

Scenarios (trip distance, type of truck)	TCO of NREL trucks [€/km]	TCO of model AHB [€/km]
575 km, diesel (NREL)	0.6108	0.6110
576 km, diesel (NREL)	0.611	0.6108

Table S12: Break-even analysis for the TCO of the model AHB and trucks powered by zero-emission technologies (BET and FCET). For the model AHB, the TCO is calculated for scenario 2 and green hydrogen. Data for the semi-trucks is taken from the 2025 weight-constrained analysis of the NREL[4].

Scenarios (trip distance, type of truck)	TCO of NREL trucks [€/km]	TCO of model AHB [€/km]
623 km, BET (NREL)	0.6007	0.6004
624 km, BET (NREL)	0.6005	0.6008

Supplementary Note 6: Life cycle inventory of the LCA

To ensure that our LCA is reproducible, the life cycle inventory (LCI) for the LCA is presented in this section. In Figure S1, a model graph of the AHB's LCI is shown. It provides an overview of the eight central processes (green) as well as some underlying process flows.

The detailed LCI for each of the eight processes (as well as an additional table for carbon fiber) is shown in the tables below. LCIs for existing and unchanged Ecoinvent processes are not broken down further.

Table S13: LCI for the aluminum hull production. This LCI deliberately excludes heat or electricity because production data for the AHB's hull is not available and therefore very prone to error. Based on own model AHB design.

	Description	Unit	Amount	Ecoinvent Process
**Input:**	Aluminum, wrought alloy	kg	10581	market for aluminum, wrought alloy/GLO
	Metal working, average for aluminum product manufacturing	kg	10581	metal working, average for aluminum product manufacturing/RER
**Output:**	*1 complete AHB hull*			

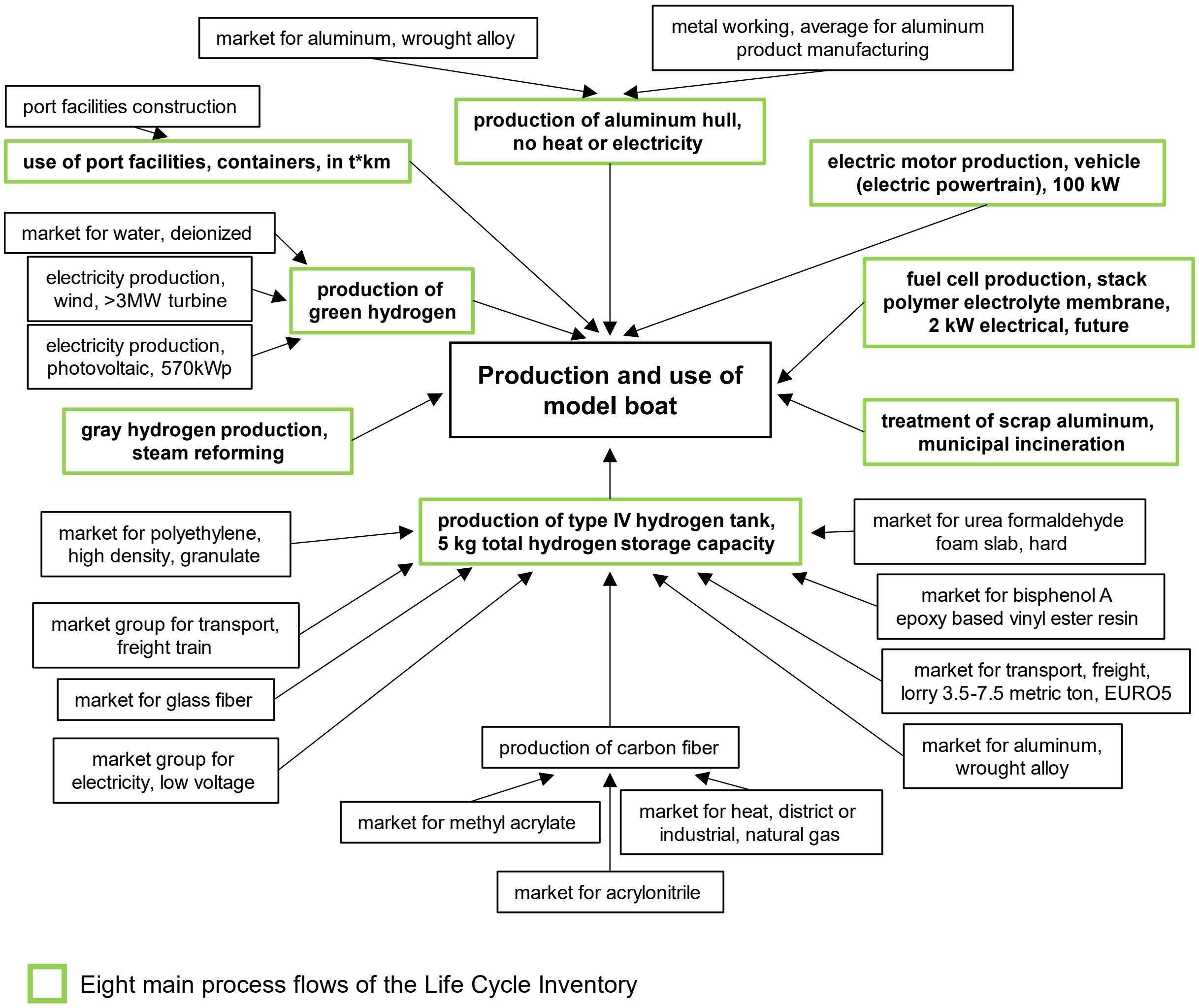

Figure S1: Model graph for the life cycle inventory of the AHB. The LCI of the model boat is based on eight main processes which are shown in green. Calculations that deliver environmental impact per km of boat travel were carried out in Excel so that values shown here are not final.

*Table S14: LCI for the production of an electric motor. Based on existing Ecoinvent process. *All processes for electricity and heat requirements were manually changed to RER.*

	**Description**	**Unit**	**Amount**	**Ecoinvent Process**
**Input:**	Electric motor production, vehicle	kg	1	electric motor production, vehicle (electric powertrain)/GLO*
**Output:**	*1 kg of electric motor (full motor: 53 kg, 100 kW)*			

*Table S15: LCI for the production of a 2 kW polymer electrolyte membrane fuel cell. Based on existing Ecoinvent process. *Process for electricity changed to RER.*

	**Description**	**Unit**	**Amount**	**Ecoinvent Process**
**Input:**	Fuel cell, stack polymer electrolyte membrane (PEMFC), 2kW electrical	Item(s)	1	fuel cell, stack polymer electrolyte membrane, 2kW electrical, future/CH*
**Output:**	*2 kW PEMFC*			

Table S16: LCI for the production of a type IV hydrogen tank with 5 kg total hydrogen storage capacity. Based on data by Usai et. al[22].

	Description	Unit	Amount	Ecoinvent Process
**Input:**	Aluminum, wrought alloy	kg	1.8	market for aluminum, wrought alloy/GLO
	Bisphenol A epoxy based vinyl ester resin	kg	25.64	market for bisphenol A epoxy based vinyl ester resin/GLO
	Electricity, low voltage	kWh	4.5	market group for electricity, low voltage/RER
	Glass fiber	kg	4.3	market for glass fiber/GLO
	Polyethylene, high density, granulate	kg	7.5	market for polyethylene, high density, granulate/GLO
	Production of carbon fiber_RER	kg	62.16	based on data by Usai *et. al*[22], also see Table S15.
	Transport, freight train	t*km	66.8	market group for transport, freight train/RER
	Transport, freight, lorry 3.5-7.5 metric ton, EURO5	t*km	11.1	market for transport, freight, lorry 3.5-7.5 metric ton, EURO5/RER
	Urea formaldehyde foam slab, hard	kg	4.3	market for urea formaldehyde foam slab, hard/GLO
**Output:**	*Type 4 hydrogen tank; 5 kg total storage capacity*			

Table S17: LCI for the production of carbon fiber. Based on data by Usai *et. al*[22].

	Description	Unit	Amount	Ecoinvent Process
**Input:**	Acrylonitrile	kg	1.64	market for acrylonitrile/GLO
	Electricity, low voltage	kWh	41.13	market group for electricity, low voltage/RER
	Heat, district, or industrial, natural gas	MJ	118.66	market for heat, district or industrial, natural gas/Europe without Switzerland
	Methyl acrylate	kg	0.09	market for methyl acrylate/GLO
**Output:**	*1 kg carbon fiber*			

Table S18: LCI for the production of gray hydrogen. Based on existing Ecoinvent process.

	Description	Unit	Amount	Ecoinvent Process
**Input:**	Gray hydrogen production, steam reforming	kg	1	hydrogen production, steam reforming/RER
**Output:**	*1 kg gray hydrogen*			

Table S19: LCI for the production of green hydrogen. A 100% renewable energy mix of equal parts photovoltaic and wind electricity is assumed. Electricity and water requirements are based on data by Al-Qahtani et. al and Saur et. al[23,24]. The electrolyzer is deliberately left out because it has a low impact compared to photovoltaic and wind electricity infrastructure [23].

	Description	Unit	Amount	Ecoinvent Process
**Input:**	Electricity, high voltage	kWh	27.15	electricity production, wind, >3MW turbine, onshore/DE
	Electricity, low voltage	kWh	27.15	electricity production, photovoltaic, 570kWp open ground installation, multi-Si/DE
	Water, deionized	kg	18.02	market for water, deionized/Europe without Switzerland
**Output:**	*1 kg green hydrogen*			

Table S20: LCI for the utilization of port facilities. This represents the environmental impact for loading and unloading containers. Based on existing Ecoinvent process but varied so that the amount fits the use case of this work.

	Description	Unit	Amount	Ecoinvent Process
**Input:**	Port facilities	Item(s)	1.38106E-16	port facilities construction/RER
**Output:**	*Loading impact per t*km of boat weight and travel*			

Table S 21: LCI for aluminum recycling. Based on existing Ecoinvent process.

	Description	Unit	Amount	Ecoinvent Process
**Input:**	Aluminum recycling (scrap aluminum)	kg	1	treatment of scrap aluminum, municipal incineration/Europe without Switzerland
**Output:**	*Emissions to the environment*			

References

1. International Container Service GmbH. 40' dry-van standard containers. https://www.icon-container.de/en/container-finder/standard-container-40-dry-van (2024).
2. Molland, A. F. *Ship Resistance and Propulsion. Practical Estimation of Ship Propulsive Power* (Cambridge University Press, 2011).
3. European Commission & Directorate-General for Environment. *Commission Recommendation (EU) 2021/2279 of 15 December 2021 on the use of the Environmental Footprint methods to measure and communicate the life cycle environmental performance of products and organisations. 32021H2279* (2021).
4. Hunter, C. *et al.* Spatial and Temporal Analysis of the Total Cost of Ownership for Class 8 Tractors and Class 4 Parcel Delivery Trucks. (National Renewable Energy Laboratory - U.S. Department of Energy, 2021).
5. Ragon, P.-L. & Rodríguez, F. CO2 emissions from trucks in the EU: An analysis of the heavy-duty CO2 standards baseline data. (International Council on Clean Transportation, 2022).
6. Hydrogen Council & McKinsey & Company. Path to Hydrogen Competitiveness: A Cost Perspective. (Hydrogen Council, 2020).
7. Kampker, A. & Heimes, H. H. *Elektromobilität. Grundlagen einer Fortschrittstechnologie* (SPRINGER VIEWEG, 2022).
8. Kleen, G., Gibbons, W. & Fornaciari, J. Heavy-Duty Fuel Cell System Cost. (US Department of Energy, 2022).
9. König, A. *et al.* An Overview of Parameter and Cost for Battery Electric Vehicles. *WEVJ* **12,** 21 (2021).
10. Bloomberg NEF. Green Hydrogen to Undercut Gray Sibling by End of Decade. https://about.bnef.com/blog/green-hydrogen-to-undercut-gray-sibling-by-end-of-decade/ (2023).
11. Dinu, O. & Ilie, A. M. Maritime vessel obsolescence, life cycle cost and design service life. *IOP Conf. Ser.: Mater. Sci. Eng.* **95,** 12067 (2015).
12. Elkafas, A. G., Rivarolo, M., Gadducci, E., Magistri, L. & Massardo, A. F. Fuel Cell Systems for Maritime: A Review of Research Development, Commercial Products, Applications, and Perspectives. *Processes* **11,** 97 (2023).
13. van Biert, L., Godjevac, M., Visser, K. & Aravind, P. V. A review of fuel cell systems for maritime applications. *Journal of Power Sources* **327,** 345–364 (2016).
14. Renault Group. All you need to know about the motor of an electric car - Renault Group. https://www.renaultgroup.com/en/news-on-air/news/learn-all-you-need-to-know-about-the-motor-of-an-electric-car/ (2024).
15. US Department of Energy. Extend the Operating Life of Your Motor. https://www.energy.gov/eere/amo/articles/extend-operating-life-your-motor (2012).

16. Ahluwalia, R., Papadias, D., Peng, J.-K. & Roh, H. System Level Analysis of Hydrogen Storage Options. (Argonne National Laboratory, 2019).

17. NPROXX. Type 4 pressure vessels for hydrogen storage - NPROXX. https://www.nproxx.com/capabilities/type-4-pressure-vessels/ (2024).

18. Rivard, E., Trudeau, M. & Zaghib, K. Hydrogen Storage for Mobility: A Review. *Materials* **12,** 1973 (2019).

19. Deng, L., Johnson, S. & Gencer, E. Environmental-Techno-Economic analysis of decarbonization strategies for the Indian aluminum industry. *Energy Conversion and Management* **274,** 116455 (2022).

20. eurostat. Glossary: Standard goods classification for transport statistics (NST). https://ec.europa.eu/eurostat/statistics-explained/index.php?title=Glossary:Standard_goods_classification_for_transport_statistics_(NST) (2023).

21. eurostat. Road freight transport by distance class and type of goods (t, tkm, vehicle-km, basic transport operations) - annual data (from 2008 onwards). https://ec.europa.eu/eurostat/databrowser/view/road_go_ta_dctg/default/table?lang=en (2023).

22. Usai, L. *et al.* Life cycle assessment of fuel cell systems for light duty vehicles, current state-of-the-art and future impacts. *Journal of Cleaner Production* **280,** 125086 (2021).

23. Al-Qahtani, A., Parkinson, B., Hellgardt, K., Shah, N. & Guillen-Gosalbez, G. Uncovering the true cost of hydrogen production routes using life cycle monetisation. *Applied Energy* **281,** 115958 (2021).

24. Saur, G., Ramsden, T., James, B. & Colella, W. Current Central Hydrogen Production from Grid PEM Electrolysis. https://www.nrel.gov/hydrogen/h2a-production-archive.html (2018).
